# Supplementary material for: Effects of pharmaceutically active compounds (PhACs) on fish body and scale shape in natural waters
Source: PeerJ. 2021 Feb 11;9:e10642. doi: 10.7717/peerj.10642 (PMC7882141; doi:10.7717/peerj.10642)
Supplement: Supplemental Information 1 — Full names of sampling points are in Fig. 1. [file peerj-09-10642-s001.docx]

**Table S1. Measured body parameters (standard length – SL, wet weight - W) and sex ratios of the sampled fish.**

Full names of sampling points are in Figure 1.

| **Species** | **Sampling point** | **SL (mm±standard deviation)** | **W (g±standard deviation)** | **Ratio of males (%)** |
| --- | --- | --- | --- | --- |
| roach | GERTOS | 72.87±7.69 | 9.27±11.28 | 23 |
| roach | SZEBIC | 86.5±10.73 | 12.5±4.38 | 13 |
| roach | TAPTAP | 98.68±23.56 | 22.4±16.47 | 57 |
| roach | TAPUJS | 116.59±14.52 | 35.85±15.42 | 63 |
| roach | TAPGYO | 108.38±18.47 | 30.15±19.76 | 62 |
| roach | TAPSZE | 81.99±21.43 | 10.84±16.14 | 44 |
| roach | TAPUJS2 | 82.26±15.86 | 11.7±14.14 | 60 |
| roach | DTCDUN | 103.25±16.28 | 28.72±15.14 | 50 |
| roach | VALBAR | 126.18±20.33 | 35.1±18.14 | 54 |
| chub | BUKIZB | 110.22±21.14 | 24.44±15.58 | 78 |
| chub | BUKSZE | 94.91±8.28 | 13.83±4.51 | 50 |
| chub | BUKTOR | 115.14±17.56 | 30.7±21.34 | 68 |
| chub | HOSKAM | 153.25±22.07 | 92±34.29 | 25 |
| chub | HOSKEL | 135.17±30.37 | 57.83±42.38 | 58 |
| chub | HOSTOR | 125.8±10.11 | 33.6±9.1 | 60 |
| chub | GOMVAC | 88.56±12.44 | 23.65±8.67 | 53 |
| chub | HOSTOR2 | 140.91±15.44 | 68.12±29.44 | 50 |
| chub | MORVER | 91.64±11.02 | 15.36±7.69 | 77 |
| gibel carp | GERCEG | 97.96±9.59 | 35.46±9.29 | 60 |
| gibel carp | GERTOR | 113±13.04 | 51.86±17.23 | 29 |
| gibel carp | HOSKEL | 103.86±10.61 | 46.57±16.48 | 57 |
| gibel carp | SZEBIC | 96.72±9.18 | 30.11±9.76 | 22 |
| gibel carp | BENBIA | 79.08±15.66 | 24.62±14.22 | 65 |
| gibel carp | HOSTOR2 | 101.68±11.82 | 46.66±17.49 | 60 |
