# Supplementary material for: Effects of pharmaceutically active compounds (PhACs) on fish body and scale shape in natural waters
Source: PeerJ. 2021 Feb 11;9:e10642. doi: 10.7717/peerj.10642 (PMC7882141; doi:10.7717/peerj.10642)
Supplement: Supplemental Information 3 [file peerj-09-10642-s003.docx]

**Table S3. Results of Mantel-tests on morphological distances (Mahalanobis distances – Md, Procrustes distances - Pd) and the differences between the significant background variables (abbreviations in Table 2 and Table 3).**

| **Species** | **Measured phenotype** | **Morphological distance** | **Background variable** | **Correlation (R)** | **p-value** |
| --- | --- | --- | --- | --- | --- |
| gibel carp | body | Md | Zn | 0.2632 | 0.50 |
| gibel carp | body | Pd | Zn | -0.3429 | 0.51 |
| gibel carp | body | Md | PROP | -0.9617 | 1 |
| gibel carp | body | Pd | PROP | -0.6263 | 0.82 |
| gibel carp | body | Md | catch.size | 0.8932 | 0.33 |
| gibel carp | body | Pd | catch.size | 0.4689 | 0.50 |
| gibel carp | scale | Md | Pb | -0.4032 | 0.71 |
| gibel carp | scale | Pd | Pb | 0.1057 | 0.46 |
| gibel carp | scale | Md | wetland | -0.5308 | 0.88 |
| gibel carp | scale | Pd | wetland | 0.1647 | 0.33 |
| chub | body | Md | Cd | -0.2236 | 0.76 |
| chub | body | Pd | Cd | -0.0925 | 0.63 |
| chub | body | Md | detritus | -0.1888 | 0.63 |
| chub | body | Pd | detritus | 0.3317 | 0.22 |
| chub | scale | Md | Mg | -0.1796 | 0.72 |
| chub | scale | Pd | Mg | 0.1414 | 0.31 |
| chub | scale | Md | As | 0.1088 | 0.35 |
| chub | scale | Pd | As | 0.2339 | 0.22 |
| chub | scale | Md | Ca | 0.7521 | 0.12 |
| **chub** | **scale** | **Pd** | **Ca** | **0.7204** | **0.01** |
| chub | scale | Md | macrophyte | 0.9036 | 0.09 |
| chub | scale | Pd | macrophyte | 0.6805 | 0.09 |
| chub | scale | Md | depth | 0.11 | 0.30 |
| chub | scale | Pd | depth | 0.01574 | 0.44 |
| chub | scale | Md | CODE | -0.1385 | 0.61 |
| chub | scale | Pd | CODE | -0.1985 | 0.50 |
| roach | body | Md | CITA | -0.3803 | 0.91 |
| roach | body | Pd | CITA | -0.1429 | 0.72 |
| roach | body | Md | TRIM | NAN | NAN |
| roach | body | Pd | TRIM | NAN | NAN |
| **roach** | **scale** | **Md** | **As** | **0.7754** | **0.03** |
| roach | scale | Pd | As | 0.7156 | 0.08 |
| roach | scale | Md | SO_4_^2-^ | 0.802 | 0.02 |
| roach | scale | Pd | SO_4_^2-^ | 0.6887 | 0.12 |
